# Unilateral Intraparotid Swelling: A Case Report of Kimura's Disease and Review of Differential Diagnosis

**DOI:** 10.1155/2013/795921

**Published:** 2013-06-04

**Authors:** N. W. Savage, V. Vucicevic Boras

**Affiliations:** ^1^Department of Oral Biology and Pathology, School of Dentistry, 200 Turbot Street, Brisbane, QLD, Australia; ^2^Department of Oral Medicine, School of Dentistry, University of Zagreb, Gunduliceva 5, 10 000 Zagreb, Croatia

## Abstract

An interesting case of Kimura's disease was described in the 42-year-old patient manifesting itself as a unilateral parotid swelling, albeit the disease usually affects both parotid glands. Furthermore, first pathohistological finding was not suggestive of the disease, revealing only fatty tissue, but on the repeated biopsy together with CT the correct diagnosis was established. It should be emphasized that Kimura's disease has to be taken into account while making differential diagnosis in parotid gland swellings, especially in people of Oriental origin.

## 1. Introduction

Kimura's disease is an uncommon finding which was first described in 1937 as a reactive, self-limiting, painless, persistent, indolent lesion mimicking neoplasm, and being of unknown etiology. Usually it is seen in young and middle-aged men (male : female = 3.5 : 1) [1]. So far, more than 60 cases with oral involvement have been reported in the literature. Primarily it affects the tissue of lymph nodes. Usually, within face and neck area, it represents itself rarely as a parotid swelling (intraparotid or paraparotid lymph nodes are in fact affected) more frequently bilateral, but also unilateral together with few cases described where palate and cheek with eyelid were involved [2, 3]. Also, a case of a patient with Kimura's disease manifesting itself as a lymphadenopathy and painful oral ulcerations was reported [4]. The common finding is also within subcutaneous tissues and lymph nodes. It can represent itself either as a single or multiple lesions, with the latter being less frequent. Other lymph nodes of the face and neck area can be affected, as well as distant subcutaneous lymph nodes either as a solitary or multiple lesions. Histopathologically, the disease is characterized with hyperplasia of the lymphoid tissue with well-developed lymphoid follicles, marked lymphocyte (eosinophil) infiltration, proliferation of thin-walled capillary venules, and varying degrees of fibrosis [1].

## 2. Case Report

**Figure 1:**
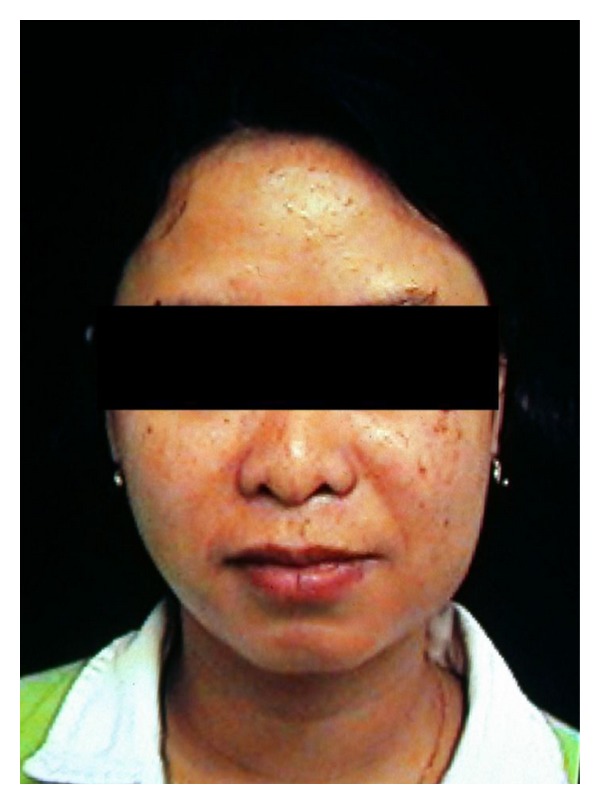
Unilateral left parotid mass.

**Figure 2:**
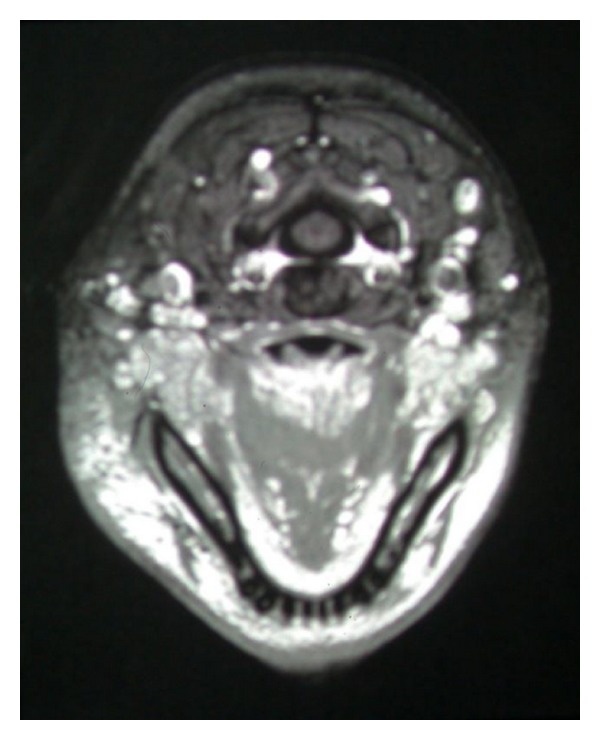
CT of the left parotid mass.

A woman, 42 years old, presented with swelling of the left parotid gland at the Department of Oral Biology and Pathology, School of Dentistry, Brisbane, Australia. The parotid nodule was painless and firm (Figure 1). OPG finding was normal and she was sent to ENT for parotid gland biopsy. Her blood test showed eosinophilia and elevated IgE levels. First histopathological finding showed only fatty tissue, then patient was sent to CT scan where mass involving parotid gland was seen (Figure 2). Again biopsy of the parotid gland was performed and this time histopathological finding revealed Kimura's disease. Parotidectomy was performed and after few years of followup the patient was well and without recurrence.

## 3. Discussion

In the past there has been a quite misunderstanding that Kimura's disease and angiolymphoid hyperplasia with eosinophilia are in fact two separate disease entities. Nowadays the difference is clear although both diseases are of unknown cause; Kimura's disease is thought to be a consequence of autoimmune/allergic response to an unknown antigen whereas angiolymphoid hyperplasia with eosinophilia is a benign vascular neoplasm [5]. However, Kimura's disease is endemic in Orientals, but of course due to increased migration trends it could be also seen in people of Oriental origin in health institutions throughout western countries. The disease can be accompanied with lymphadenopathy and pruritus on the lower limbs although manifesting itself in the head and neck region [6].

Macroscopic differential diagnosis should include chronic inflammatory diseases of the salivary glands such as recurrent bacterial infections, granulomatous gland diseases (cat-scratch disease, sarcoidosis), and autoimmune ones (Sjögren Syndrome). Furthermore, benign lymphoepithelial lesion, parotid mucocele, Warthin's tumor, and all the other benign and malignant tumors and cysts must be differentiated [7–9]. Additionally differential diagnosis might include lymphoma, metastases, angiolymphoid hyperplasia with eosinophilia [8] as well as Langerhans cell histiocytosis, florid follicular hyperplasia, Castleman disease, drug reaction, dermatopathic lymphadenopathy, parasitic lymphadenitis, and allergic granulomatosis of Churg and Strauss [6].

It has been reported that diagnosis is difficult to attain without a biopsy and CT scan is helpful due to lesion enhancement as a result of increased vascularity which “lights up” with the contrast dye. However, we do highlight the fact that even after biopsy sometimes the histopathological finding is not typical suggesting that repeated biopsy should be performed in uncertain cases. This has been also confirmed by the report of Iida et al. [2].

Although the lesion is probably immunologically related, corticosteroids are partly successful and surgical excision is recommended, whilst radiation and cytotoxic therapy are reserved for the refractory lesions not responding to the surgery.

To our knowledge this is the second case report describing Kimura's disease in the parotid gland.

This case report highlights the necessity of taking into account Kimura's disease when differentiating between salivary gland swelling especially in Oriental people.
